# Trends and disparities in mortality due to hypertensive disease and coexisting obesity in the USA: 1999–2023

**DOI:** 10.1186/s43044-025-00677-5

**Published:** 2025-08-26

**Authors:** Asad Gul Rao, Amna Parvez, Sufyan Shahid, Neha Pervez, Jamal S. Rana, Marat Fudim, Muhammad Shahzeb Khan

**Affiliations:** 1 Dow University of Health Sciences, Karachi, Pakistan; 2 Liaquat National Hospital, Karachi, Pakistan; 3 Khawaja Muhammad Safdar Medical College, Sialkot, Pakistan; 4 Kaiser Permanente Northern California, Oakland, USA; 5 Duke University Medical Center, Durham, USA; 6 Baylor Scott and White Research Institute Cardiac Imaging Core Laboratory, Plano, USA; 7 Baylor Scott and White Heart Hospital, Plano, USA

**Keywords:** Obesity, Hypertensive disease, USA, Mortality, Disparities, Epidemiology

## Abstract

**Background:**

Hypertensive disease and obesity frequently coexist and synergistically increase the risk of cardiovascular morbidity and mortality in the USA. Despite this intersection, national trends and disparities in mortality attributable to both conditions remain underexplored.

**Methods:**

We conducted a retrospective analysis using the Centers for Disease Control and Prevention Wide-ranging Online Data for Epidemiologic Research Multiple Cause of Death database. Hypertensive disease-related deaths with co-listed obesity were extracted for US adults between 1999 and 2023. Age-adjusted mortality rates (AAMRs) were calculated, and Joinpoint regression was used to estimate annual percentage changes (APCs) and identify significant trends.

**Results:**

A total of 412,767 deaths were attributed to hypertensive disease and coexisting obesity from 1999 and 2023. The AAMRs rose nearly tenfold, from 1.3 per 100,000 in 1999 to 13.23 in 2023. While mortality rates increased overtime for both sexes, men consistently exhibited higher rates than women (AAPC: 10.38 vs. 8.15). Older adults (AAMR: 32.63) had the highest mortality, followed by middle-aged (5.56) and young adults (0.71), though young adults saw the steepest relative rise (AAPC: 9.63). Non-Hispanic (NH) Black individuals had the highest mortality (AAPC: 7.78), followed by NH American Indian/Alaska Native (8.76), NH White (9.90), Hispanic (6.48) and NH Asian/Pacific Islander populations (6.51). Geographic disparities widened over time. The South and Midwest bore the heaviest regional burden, while urban–rural analyses showed a higher and quicker rise in mortality in non-metropolitan areas (AAPC: 12.13 vs. 10.73 in metro areas).

**Conclusion:**

Mortality due to hypertensive disease with coexisting obesity has escalated sharply across USA over the past 2 decades. These results highlight the need for further investigation into the factors contributing to the observed disparities and trends.

**Graphical abstract:**

Mortality due to hypertensive disease and coexisting obesity in the USA: 1999–2023
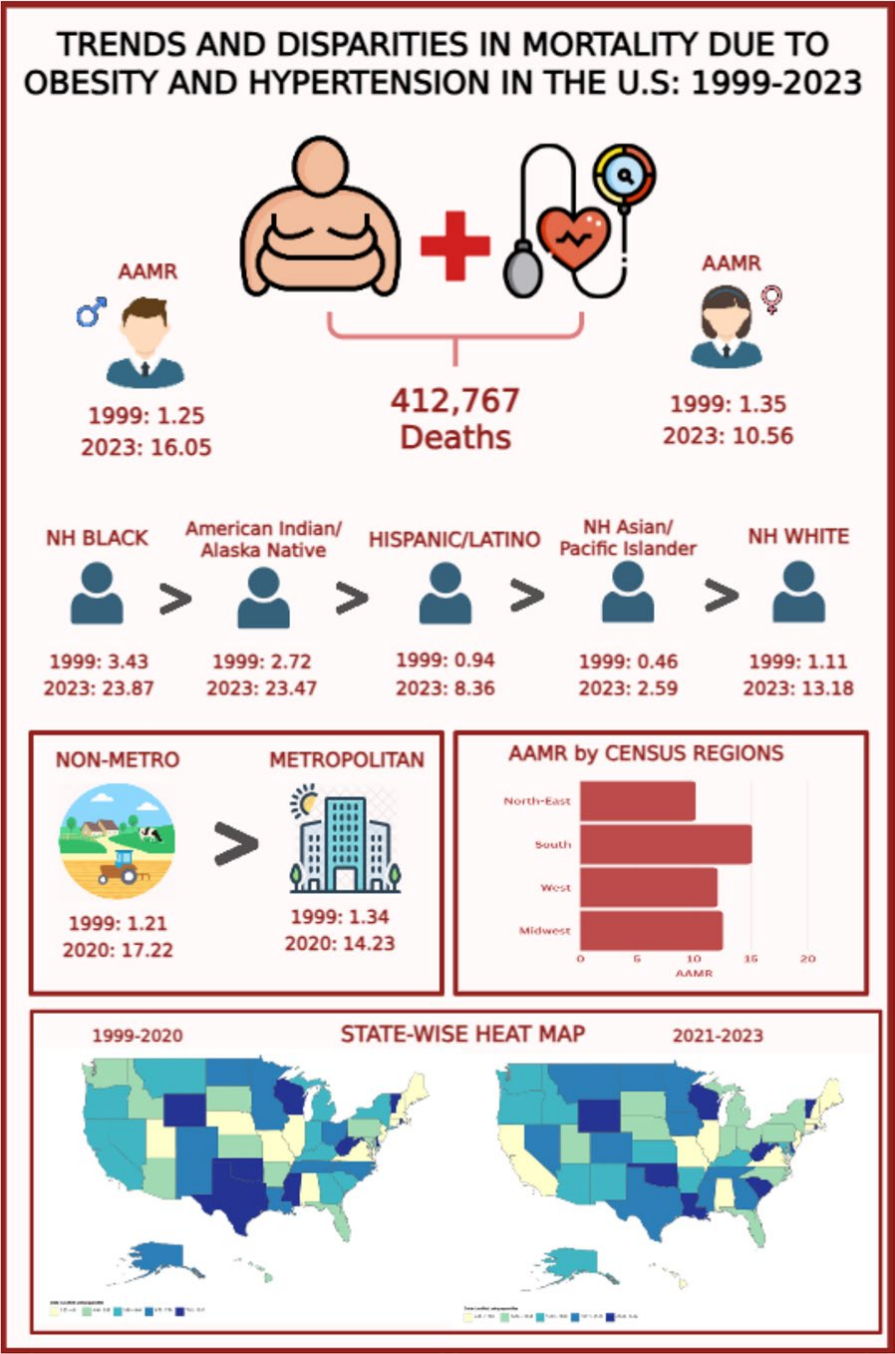

**Supplementary Information:**

The online version contains supplementary material available at 10.1186/s43044-025-00677-5.

## Introduction

Obesity, once only considered a lifestyle problem, has now emerged as a global public health crisis as it contributes to millions of preventable deaths worldwide. As of 2022, an estimated 890 million adults were obese globally, with 3.7 million deaths linked to associated non-communicable diseases (NCDs) such as cardiovascular disease, diabetes and chronic kidney and liver disorders [1]. In the United States (USA) alone, approximately 500,000 deaths each year are attributed to obesity-related causes [2].

A major contributor to this burden is the close relationship between obesity and hypertensive disease. Excess adiposity, particularly visceral fat, accounts for up to 75% of the risk of developing hypertensive disease in adults [3]. Although almost half of American adults (47.7%) suffer from hypertensive disease, its effects are frequently exacerbated when it coexists with obesity [4]. The mechanisms linking these two conditions include sympathetic nervous system over-activation, insulin resistance, baroreceptor dysfunction and activation of the renin–angiotensin–aldosterone system [5]. These pathways result in vascular and renal changes that make obesity-induced hypertensive disease harder to manage and more deadly [5]. Notably, hypertensive disease has an annual economic cost of $131 billion in the USA, further increasing the societal impact of this disease cluster [6].

In the USA, the recent studies reveal regional disparities in obesity-related mortality, with the majority of deaths occurring in Southern and Midwestern states [2, 7]. Emerging evidence also points to a similar pattern in mortality linked to hypertensive disease, suggesting a synergistic mortality burden in which obesity may be the primary driver [7]. Both conditions also disproportionately affect certain demographic groups including men and non-Hispanic (NH) Black populations, highlighting the role of structural and socioeconomic determinants of health [2, 7].

While prior studies have largely examined mortality trends related to obesity and hypertension as standalone conditions, few have focused on their co-occurrence as a compounded mortality risk. Given their shared pathophysiology and shared risk profile, a combined analytical approach may offer deeper insights into their epidemiologic and public health burden. This study diverges from conventional analyses by framing obesity not only as a chronic disease but also as a potent modifier of hypertensive mortality. Therefore, we aim to analyze national mortality data from 1999 to 2023 to examine temporal trends, geographic variations and demographic disparities in deaths attributable to hypertensive disease and coexisting obesity among US adults. By identifying trends in this intersectional disease burden, we hope to better inform prevention efforts, clinical prioritization and targeted public health interventions.

## Methods

### Study design and population

This study examined national mortality trends involving both hypertensive disease and coexisting obesity in the USA over a 25-year period from 1999 to 2023. Data were obtained from the CDC Wide-Ranging Online Data for Epidemiologic Research (CDC WONDER) [8], which compiles non-identifiable death certificate records from all 50 states and the District of Columbia. This database is widely used in epidemiological studies for its standardized and comprehensive reporting of mortality statistics across diverse population groups. The analysis included individuals aged 25 years and older whose death certificates listed both hypertensive disease and obesity as either the underlying or contributing causes of death. Through the Multiple Cause of Death Public Use Record, deaths were identified through the International Classification of Diseases, Tenth Revision (ICD-10), with code E66 representing obesity and I10–I15 representing hypertensive diseases. Additionally, we adhered to the guidelines set forth by the Strengthening the Reporting of Observational Studies in Epidemiology (STROBE) reporting criteria [9]. As the dataset is publicly available and anonymized, the study was exempt from institutional review board (IRB) review.

### Data extraction

The dataset comprised annual population counts and demographic characteristics including sex, age, race and ethnicity, geographic region, level of urbanization and place of death. Racial and ethnic categories followed the CDC WONDER classification scheme and included Hispanic or Latino, NH White, NH Black or African American and a combined group of other NH populations, such as Asian or Pacific Islander, Native Hawaiian, American Indian or Alaska Native. Age was grouped into three brackets: 25–44 years (young adults), 45–64 years (middle-aged adults) and 65 years and older (older adults). Geographic location was determined using US Census Bureau regional divisions, which categorize the USA into the Northeast, Midwest, South and West [10]. Urban–rural status was determined using the National Center for Health Statistics Urban–Rural Classification Scheme, which, based on the 2013 US Census definitions, categorized urban areas as large or medium/small metropolitan counties and rural areas as counties with populations under 50,000 [11]. Place of death was recorded in the dataset and categorized into hospitals, nursing facilities, private homes, hospice centers or other/unspecified settings. All available data were incorporated into the analysis without any omissions, exclusions or data suppression.

### Statistical analysis

To assess mortality trends linked to hypertensive disease and coexisting obesity, both crude mortality rates (CMRs) and age-adjusted mortality rates (AAMRs) were calculated per 100,000 population annually from 1999 to 2023. CMRs were derived by dividing the total number of relevant deaths by the US population for each year. AAMRs were calculated using the 2000 US standard population for age adjustment, and 95% confidence intervals were reported for all estimates [12]. These metrics enabled comparison across different demographic subgroups and over time. Temporal changes in AAMRs were evaluated using Joinpoint Regression Program (Version 5.1.0, National Cancer Institute), which identifies inflection points and calculates annual percentage change (APC) with corresponding 95% confidence intervals [13]. A two-tailed *p* value of < 0.05 was considered statistically significant. Log-linear regression models were applied to assess the direction and significance of trends, with slopes differing from zero indicating meaningful increases or decreases.

## Results

Between 1999 and 2023, a total of 412,767 deaths occurred in patients with hypertensive disease and coexisting obesity (Supplemental Table 1). Among the 386,932 cases with a recorded place of death, 48.99% occurred at home, 44.02% in medical facilities, 5.77% in nursing homes or long-term care facilities and 1.28% in hospices (Supplemental Table 2).

### Demographic trends in mortality

**Fig. 1:**
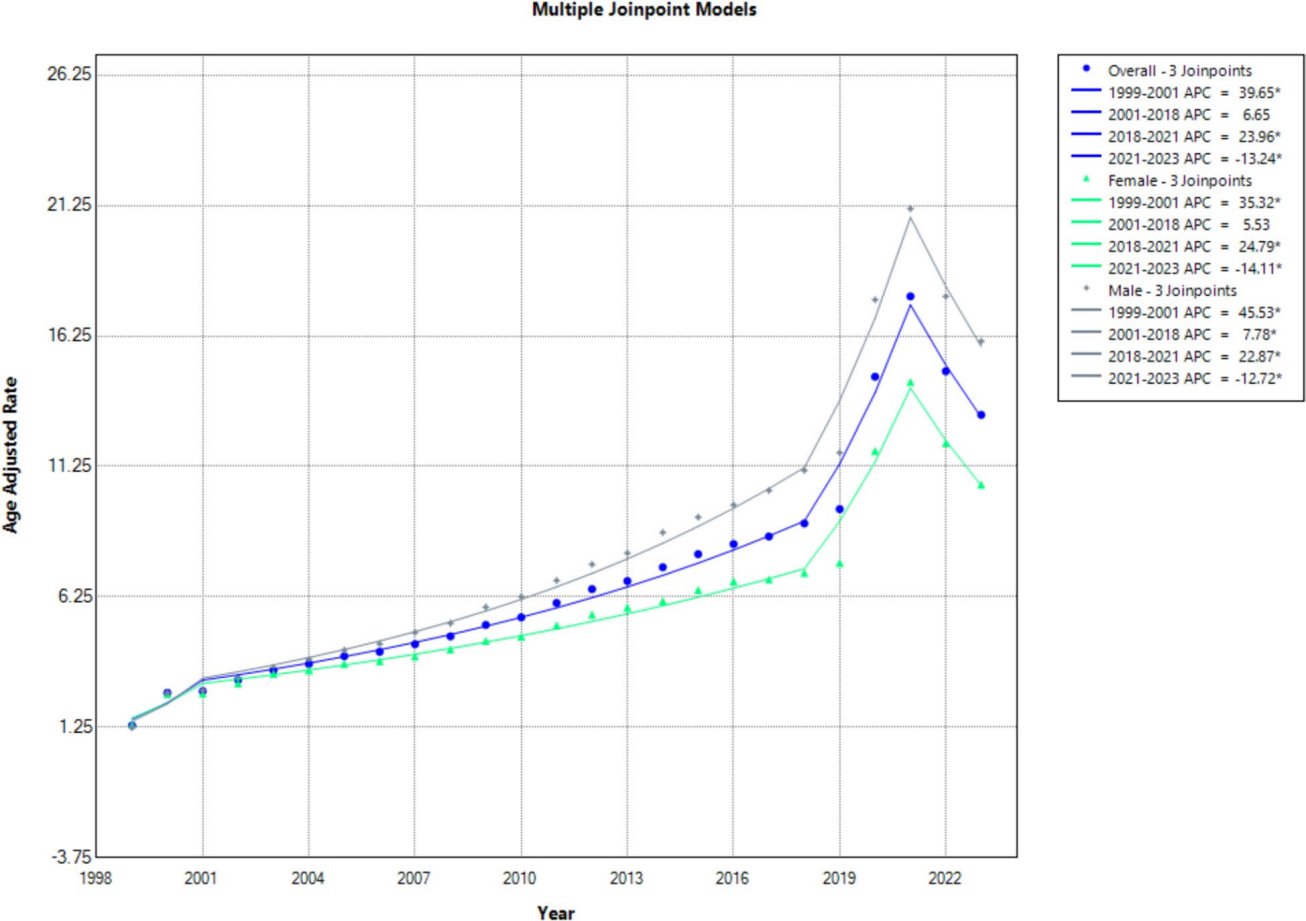
Overall and sex-stratified age-adjusted mortality rates per 100,000 for hypertensive disease and coexisting obesity in the USA, 1999 to 2023

The AAMR for patients with hypertensive disease and coexisting obesity increased markedly over the study period, rising from 1.3 per 100,000 in 1999 to 13.23 in 2023. Mortality trends showed substantial variation over time. A sharp increase was observed from 1999 to 2001 (APC: 39.65; 95% CI 8.16–79.65; *p* < 0.001), followed by a non-significant upward trend from 2001 to 2018 (APC: 6.65; 95% CI − 0.39 to 7.46; *p* > 0.05). Mortality then rose sharply from 2018 to 2021 (APC: 23.96; 95% CI 16.86–28.38; *p* < 0.001) before significantly declining between 2021 and 2023 (APC: − 13.24; 95% CI − 20.02 to − 6.27; *p* < 0.001) (Fig. 1, Supplemental Tables 3 and 4).

### Age and sex disparities

Men consistently exhibited higher mortality rates than women throughout the study period, with AAPCs of 10.38 and 8.15, respectively. Among men, mortality rose sharply from 1999 to 2001 (APC: 45.53; 95% CI 10.15–87.60; *p* < 0.001), continued to increase significantly from 2001 to 2018 (APC: 7.78; 95% CI 2.84–8.60; *p* < 0.001), peaked between 2018 and 2021 (APC: 22.87; 95% CI 16.40–26.94; *p* < 0.001) and then declined from 2021 to 2023 (APC: − 12.72; 95% CI − 19.19 to − 6.27; *p* < 0.001). Women followed a similar pattern: a significant surge from 1999 to 2001 (APC: 35.32; 95% CI 7.01–68.65; *p* < 0.001), a non-significant trend from 2001 to 2018 (APC: 5.53; 95% CI − 1.63 to 6.31; *p* > 0.05), a sharp rise from 2018 to 2021 (APC: 24.79; 95% CI 17.13–29.61; *p* < 0.001) and a significant decline from 2021 to 2023 (APC: − 14.11; 95% CI − 21.26 to − 6.87; *p* < 0.001) (Fig. 1, Supplemental Tables 3 and 4).

Age stratification revealed that older adults experienced the highest overall mortality rates (AAPC: 7.86; 95% CI 7.17–8.67), followed by middle-aged (AAPC: 8.89; 95% CI: 7.19–11.11) and young adults (AAPC: 9.63; 95% CI 8.85–10.82). Among young adults, mortality increased sharply from 1999 to 2001 (APC: 44.38; 95% CI 23.97–64.13; *p* < 0.001), rose steadily from 2001 to 2015 (APC: 8.42; 95% CI 7.79–9.51; *p* < 0.001), stabilized from 2015 to 2018 (APC: 1.44; 95% CI − 1.62 to 5.35; *p* > 0.05), spiked from 2018 to 2021 (APC: 23.06; 95% CI 18.68–27.08; *p* < 0.001) and then declined sharply (APC: − 15.03; 95% CI − 19.64 to − 10.76; *p* < 0.001). Middle-aged adults experienced a similar pattern, with an initial surge from 1999 to 2001 (APC: 43.08; 95% CI 8.59–82.74; *p* < 0.001), a slower increase through 2018, a spike during 2018–2021 and a decline thereafter. Older adults saw a steady increase until 2018, a sharp rise from 2018 to 2021 (APC: 27.30; 95% CI 19.85–31.78; *p* < 0.001) and a significant decline from 2021 to 2023 (APC: − 11.18; 95% CI − 17.94 to − 3.89; *p* < 0.001).

**Fig. 2:**
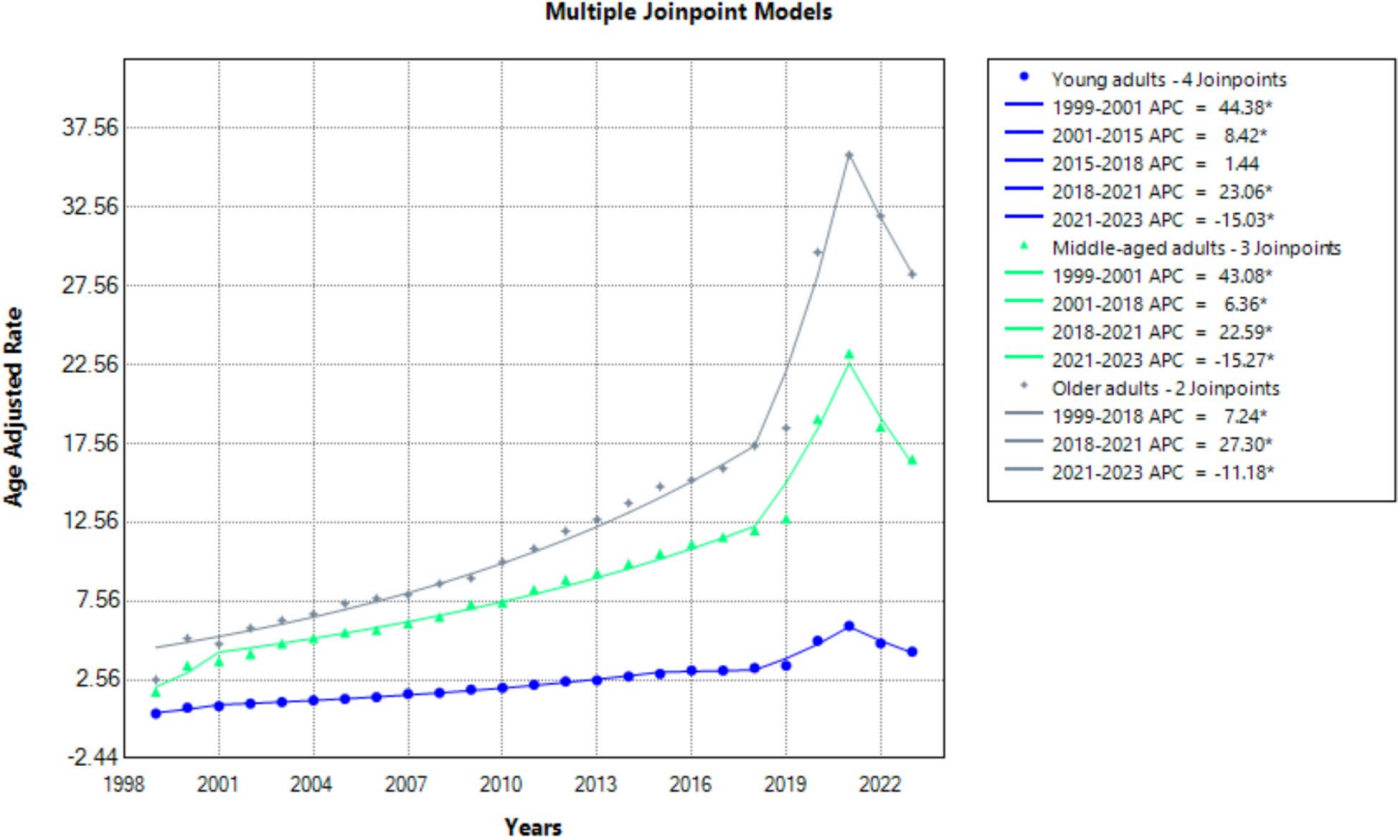
Age-adjusted mortality rates per 100,000 for hypertensive disease and coexisting obesity, stratified by age in the USA, 1999 to 2023

Absolute AAMRs were highest in older adults (32.63), followed by middle-aged (5.56) and young adults (0.71). Notably, all age groups exhibited a consistent pattern of decline from 2021 to 2023 following a 2018–2021 surge (Fig. 2, Supplemental Tables 3 and 5).

### Racial and ethnic inequities

**Fig. 3:**
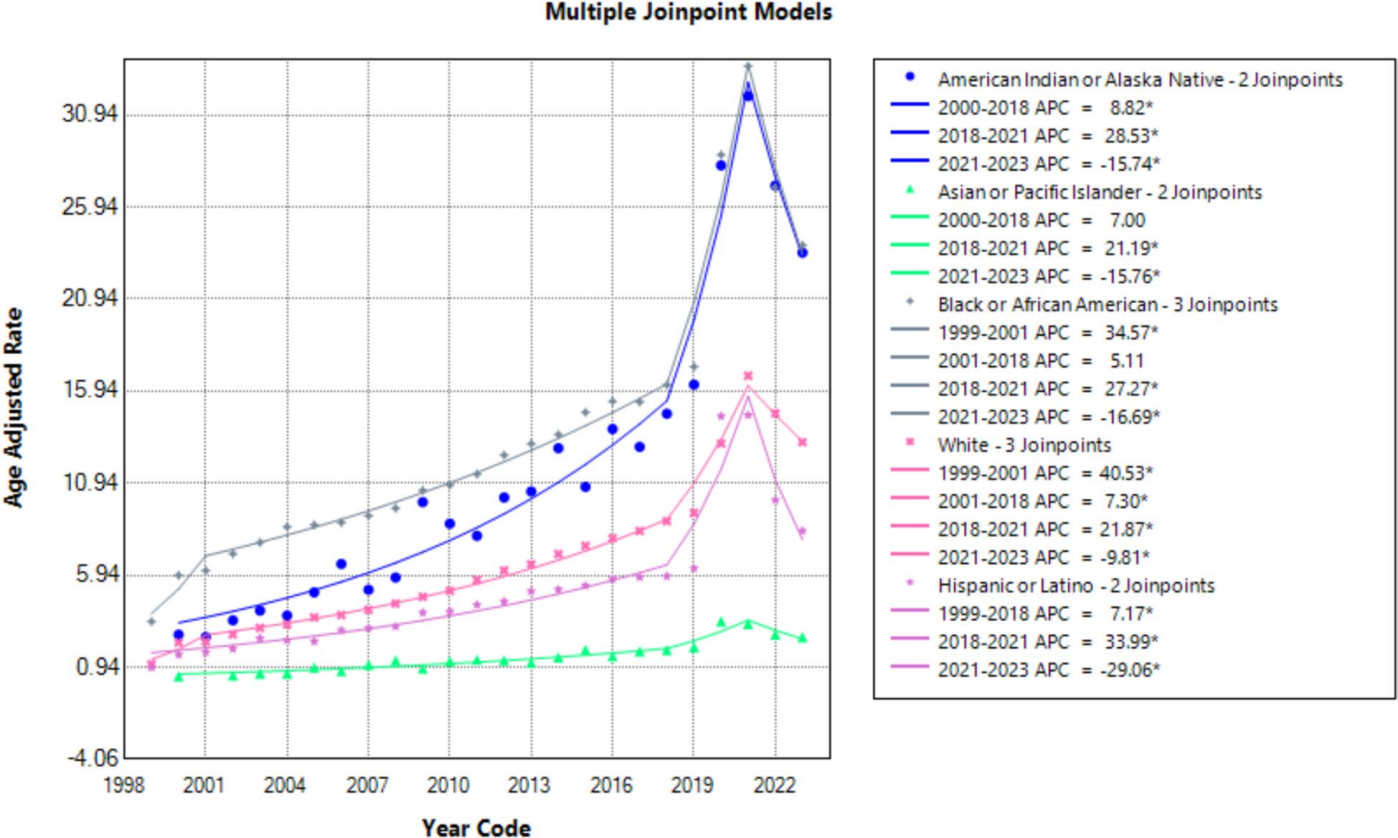
Age-adjusted mortality rates per 100,000 for hypertensive disease and coexisting obesity, stratified by census regions in the USA, 1999 to 2023

Racial disparities in AAMRs were prominent. NH Black individuals had the highest AAMRs (AAPC: 7.78; 95% CI 6.20–9.78), followed by NH American Indian/Alaska Native (AAPC: 8.76), NH White (AAPC: 9.90), Hispanic (AAPC: 6.48) and NH Asian/Pacific Islander populations (AAPC: 6.51). Most racial groups demonstrated rising trends until 2018, a spike during 2018–2021 and a decline afterward. NH American Indian/Alaska Native individuals experienced a pronounced increase through 2021, followed by a sharp decline (APC: − 15.74; 95% CI − 25.83 to − 3.18; *p* < 0.001). Similar post-2021 declines were observed across NH Asian/Pacific Islander, NH Black, Hispanic and NH White populations (Fig. 3, Supplemental Tables 3 and 6).

### Geographic and urban–rural patterns

From 1999 to 2020, AAMRs showed marked geographic disparities, ranging from 3.23 to 4.56 in the lowest 10% of states (Connecticut, Alabama, Virginia, Massachusetts, Missouri, Nebraska) to 8.56–15.11 in the highest (West Virginia, Delaware, Mississippi, District of Columbia, Oklahoma, Vermont), reflecting a two- to fivefold difference.

In 2021–2023, disparities widened. The lowest rates were observed in Massachusetts, Virginia, New Jersey, Connecticut, Hawaii and Maine (7.56–9.80), while Vermont, Wyoming, Rhode Island, Delaware, South Carolina and Oklahoma had the highest (26.01–47.42), indicating a three- to sixfold difference. Persistent high-burden states included West Virginia and Oklahoma, while new hotspots emerged in Wyoming and Rhode Island (Supplemental Table 7A).

**Fig. 4:**
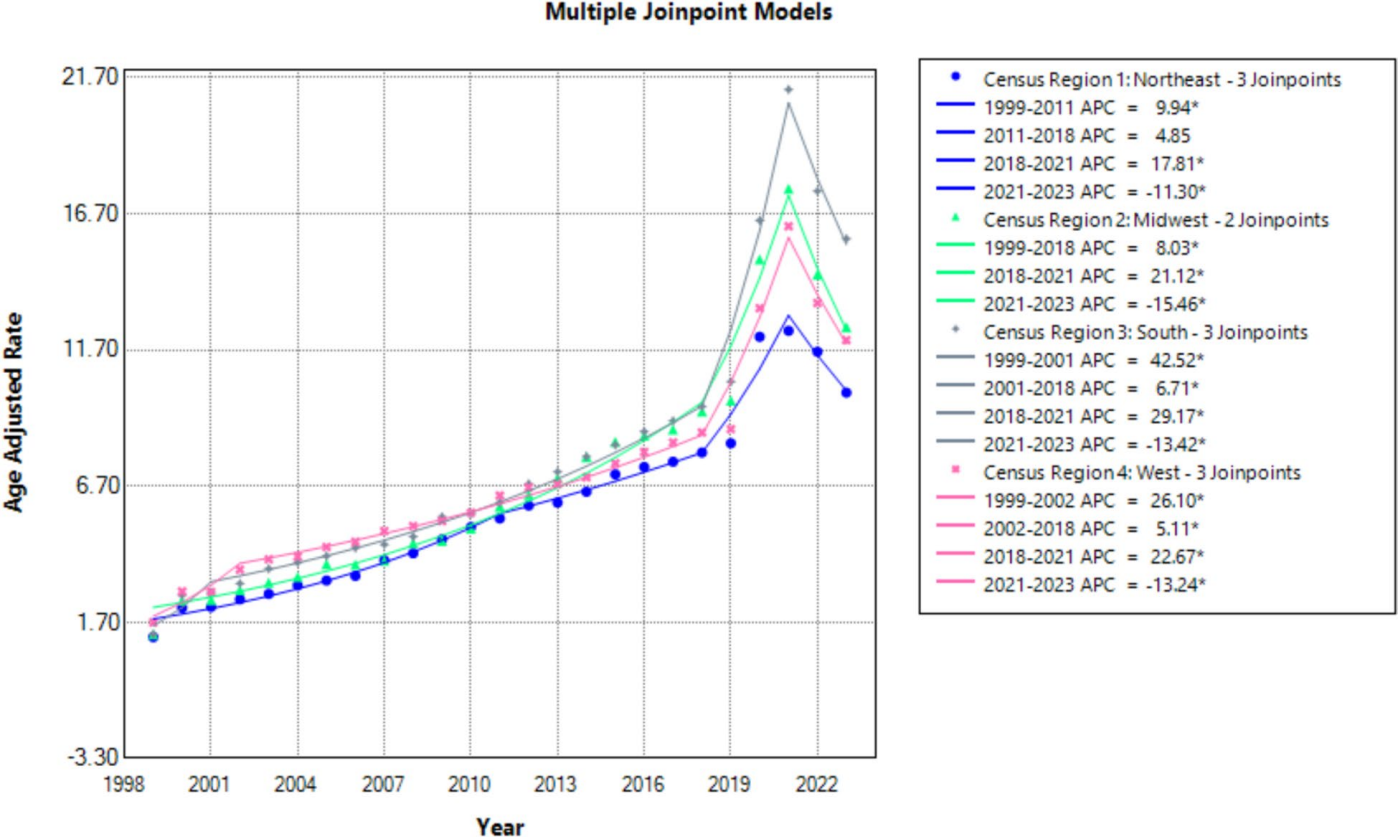
Age-adjusted mortality rates per 100,000 for hypertensive disease in obese patients, stratified by census regions in the USA, 1999 to 2023

Regionally, the Northeast experienced a steady increase from 1999 to 2011, a plateau until 2018, followed by a surge during 2018–2021 and a decline thereafter. The Midwest showed a continuous rise until 2018, a spike during 2018–2021 and a post-2021 decline. The South exhibited the steepest early rise from 1999 to 2001, a continued increase until 2018, a surge in 2018–2021 and a decline from 2021 to 2023. The West followed a similar trend, with a particularly steep rise in the early years and another sharp increase from 2018 to 2021 before declining (Fig. 4, Supplemental Tables 3 and 8).

**Fig. 5:**
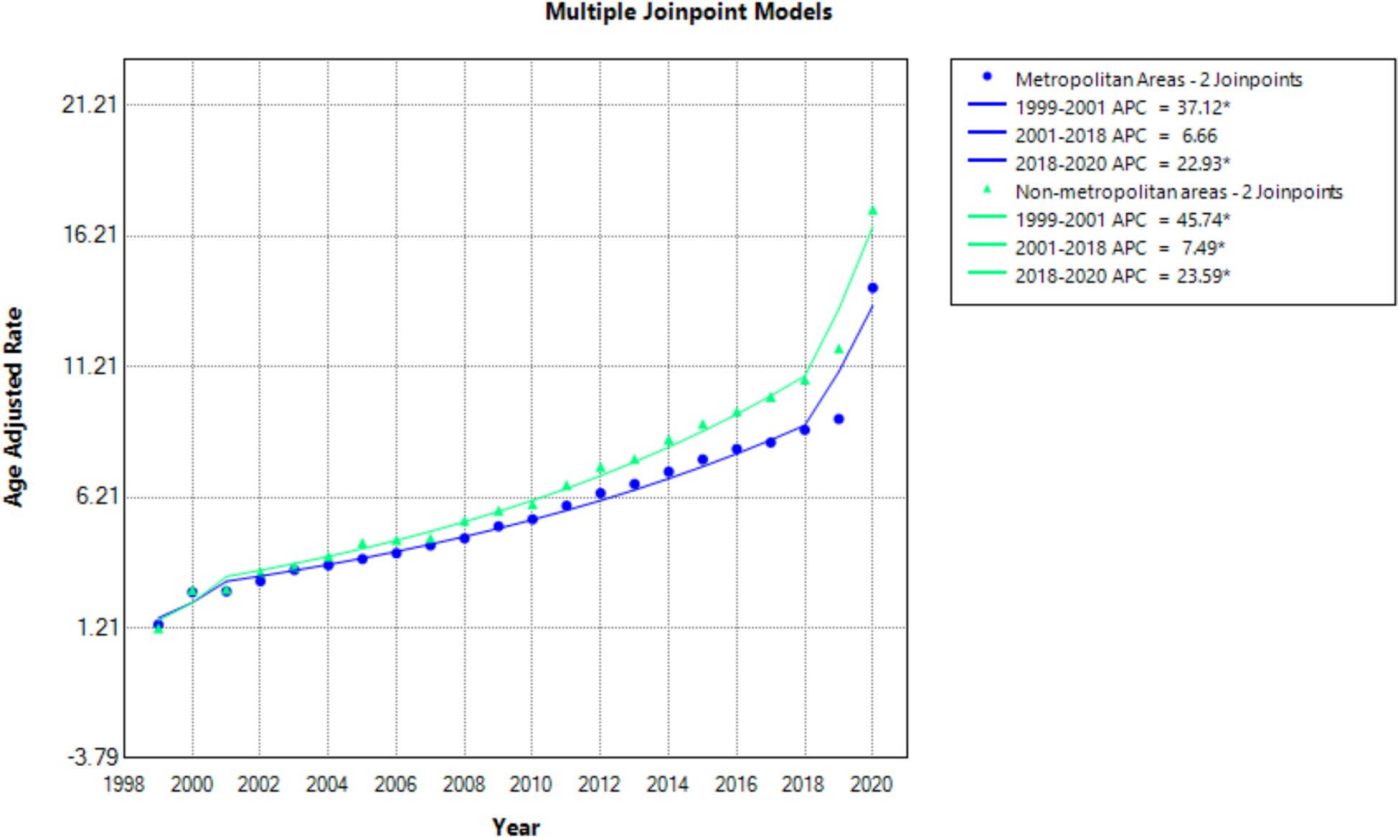
Age-adjusted mortality rates per 100,000 for hypertensive disease and coexisting obesity in the metropolitan and non-metropolitan areas in the USA, 1999 to 2020

Urban–rural mortality disparities were analyzed for 1999–2020 due to unavailable data beyond 2020. Non-metropolitan areas consistently experienced higher mortality than metropolitan regions, with AAPCs of 12.13 and 10.73, respectively. In metropolitan areas, mortality surged from 1999 to 2001, remained stable until 2018 and then spiked during 2018–2020. Non-metropolitan areas saw a similar pattern but with more pronounced increases during each time interval, particularly from 2001 to 2018 (APC: 7.49; 95% CI 6.20–8.17; *p* < 0.001) and again from 2018 to 2020 (APC: 23.59; 95% CI 13.86–29.29; *p* < 0.001) (Fig. 5, Supplemental Tables 3 and 9).

## Discussion

This nationwide study examined long-term mortality trends related to hypertensive disease and coexisting obesity among US adults aged 25 and older using CDC WONDER data from 1999 to 2023. The AAMRs rose tenfold during this period, with a particularly steep spike between 2018 and 2021. Although mortality rates have declined since 2021, reflecting the resumption of routine health care after COVID-19 pandemic, the overall trajectory of mortality due to hypertensive disease and coexisting obesity over the last 2 decades remains upward.

Our analysis showed a substantial variation in mortality trends over the past 2 decades. The early surge in AAMR from 1999 to 2001 reflects fast food industry growth, advent of the obesity epidemic and the distinct public health priorities of that period [14–16]. A rise in obesity and its associated cardiovascular complications were observed following an increase in processed food consumption, reduced physical activity and relevant technological advancements of the time [15, 17]. Additionally, obesity-related hypertensive disease mortality increased sharply from 2018 to 2021. These particular years were heavily impacted by the COVID-19 pandemic, and all non-essential healthcare services were delayed or halted across the country [18]. This led to an increased mortality observed in adult hypertensive patients during this time, due to interruptions in routine healthcare checkups and delay in seeking care [19]. Notably, the SARS-CoV-2 virus itself contributed significantly to cardiovascular complications in patients worldwide [20]. The pandemic era was intensified with a rise in sedentary lifestyles, mental health disorders, obesity and a delay in care seeking mentality driven by fears of exposure and skepticism toward overburdened healthcare institutions [21–24]. The declining pattern observed in the three years after the pandemic could be a reflection of resumption of routine healthcare services and advancements made in telemedicine and remote monitoring devices [25].

The aging population of the USA displayed the highest AAMRs across all stratified age groups. This is consistent with the increased cardiovascular risk associated with obesity in older adults, causing high mortality at similar BMIs compared to younger cohorts [26]. An age-related decline in autonomic regulation, kidney function and vascular health, combined with chronic inflammation, comorbid conditions and poor medical compliance, all contribute to the high mortality observed in this group [27]. However, many societal and infrastructural limitations also increase risk in these vulnerable populations. Older adults require coordinated care for multiple comorbidities and polypharmacy, often unavailable at most clinics [28]. Their financial constraints further limit their availability of nutritious food and recreational activity [29]. Clinically, older adults experience reduced rates of therapy intensification for BP control, limited insurance coverage and are poor candidates for medical or surgical interventions for weight loss [27, 30, 31]. Conditions like obstructive sleep apnea (OSA), dyslipidemias and depression in the elderly further compound their risk for developing hypertensive disease [32, 33]. While young adults ≥ 25 years had the lowest AAMRs, the observed rise is concerning when considering their low baseline levels. Recently, Tang et al. reported a concerning decline in hypertensive disease awareness, treatment and control in the young adult population of the USA [34]. Given the parallel rise of obesity in this age group, early intervention may be deemed necessary as young adults often delay seeking health care, leading to late initiation of crucial antihypertensive therapy and lifestyle modifications that could mitigate long-term cardiovascular damage [26, 35]. The increased prevalence of a sedentary lifestyle, primarily driven by increased screen time and urban infrastructure, further contributes to poor cardiometabolic health in this population [36]. Modifiable factors such as excess fast food consumption, high sodium diet, environmental stressors and poor sleeping habits are common driving forces for both obesity and hypertensive disease in young adults [36]. Notably, mental health diseases like binge-eating disorders, anxiety and depression also continue to rise in this population, further exacerbating their risk [37]. Finally, the observed rise in hypertensive disease and coexisting obesity in the US adult population suggests a complex pathophysiological interplay, possibly influenced by regional policy differences and unmet preventive care needs.

Gender disparities identified in our analysis not only highlight distinct biological characteristics but also mirror the deeply ingrained societal norms and gender-specific struggles. Mortality due to hypertensive disease and coexisting obesity was most prevalent in the US adult male population, despite comparable or lower incidence rates of these conditions in men [1, 38]. Simultaneously, a higher fatality burden of obesity and hypertensive disease is placed on the male population [7, 26]. Although women are more likely to be inflicted with mental health and endocrine disorders commonly associated with obesity, men are more likely to engage in alcoholism, smoking and recreational drug use [39, 40]. Furthermore, the male body has an increased tendency to accumulate visceral fat, a primary driver of insulin resistance and inflammation [41]. Incidentally, testosterone in men has been linked to increased sympathetic nervous system activation and high blood pressure [42]. In contrast, estrogen in women provides a certain degree of vascular protection [43]. Beyond biological differences, societal norms may further compound these observed gender disparities with conventional norms around ‘masculinity’ often discouraging health-seeking behavior in men. Traditional gender roles discourage men in expressing vulnerability and engaging in preventive metabolic screening [44]. Male dominated jobs often require physical labor but are accompanied with a high stress environment and irregular work hours, promoting unhealthy eating patterns and providing decreased time for personal health [44]. Excess weight is arguably less stigmatized in men, with women facing increased societal pressure from a young age to look a certain way [45]. Consequently, fat loss regimens and advertisements are mostly designed for the female audience with less focus on their male counterparts [46]. Conventionally, excess weight is generally considered more detrimental in women, largely due to fertility concerns, with male fertility effects receiving less attention in comparison [47]. Despite these recognized differences, a uniform approach is taken in the identification and management of obesity-related hypertensive disease, with the exception of pregnancy-related hypertensive disease. Currently, no sex- or gender-specific standards of care are established in the American College of Cardiology/American Heart Association guidelines [48].

The elevated mortality observed in the NH Black population of the USA is driven by multiple variables. A complex interaction of biological factors, racial segregation, higher rates of obesity, limited access to preventive healthcare and long-standing structural inequalities contribute to the rising discrepancy noted in this population [49]. There are several pathophysiological factors that identify this population at particular risk of developing adverse complications of hypertensive disease [49]. However, African Americans are still less likely to be enrolled in hypertensive disease clinical trials and receive inadequate preventive counseling sessions [50]. NH Black men display a general lack of trust in health care and are significantly more likely to delay routine checkups, blood pressure monitoring and cholesterol screenings [44]. Economic instability, racial bias, limited healthcare access and lower literacy rates in African American communities further compound their risk [44, 49, 50]. Furthermore, lower-middle income and segregated communities like the NH blacks are more likely to be living in a food swamp, with increased access to fast food, processed goods and alcoholic beverages [51–53]. The NH Asian/Pacific Islander population is a close runner up in mortality due to hypertensive disease and coexisting obesity. This population observes an under-recognized cardiometabolic risk at lower BMIs, low health literacy in elders, cultural stigma and significant language barriers [54]. In Asians, WHO recommends lower cutoffs for characterizing overweight (BMI of ≥ 23.0 kg/m^2^) and obese (BMI ≥ 25.0 kg/m^2^) categories [54]. This disparity outlines the need for provisional recommendations to be revised and tailored in light of further validation of studies and clinical experience.

There were several geographic disparities noted in mortality due to hypertensive disease and coexisting obesity in the USA, likely a reflection of persistent state-level policy gaps in Medicaid expansion and preventive care strategies. Compared to the South, the Northeastern USA experienced a significant plateau in mortality following The Affordable Care Act (ACA) implementation, expanding health coverage and preventing insurers from denying coverage based on preexisting/chronic conditions [55]. Better public health engagement and sound healthcare policies likely contributed to an early plateau and quick post-pandemic recovery of mortality in these regions. Northeastern states with established obesity prevention programs, improved healthcare infrastructure and Medicaid expansion like Virginia, Massachusetts, New Jersey, Connecticut, exhibited the lowest AAMRs. Despite broader insurance coverage, Rhode Island emerged as a hotspot for obesity-related hypertensive disease mortality, likely driven by rising obesity rates and undetermined socioeconomic variables [56]. The Southern USA displayed some of the highest AAMRs, with West Virginia, Oklahoma, South Carolina and Mississippi bearing the majority of this mortality burden. These trends are the reflection of rising rates of obesity, poverty and alcohol along with limited insurance coverage continually impacting public health in these regions [56]. Trends in the West provided an assorted risk assessment profile with progressive public health policies counterbalanced by rural healthcare gaps. States like Wyoming are found to be at emerging risk with a rapid rise in obesity rates and stagnant preventive efforts requiring urgent attention.

The raised AAMR observed in non-metropolitan areas signifies a persistent socioeconomic disadvantage still faced by rural populations [57]. Inequality of medical resources, limited specialist availability and financial barriers prevent routine healthcare checkups, medication adherence and thorough chronic disease management in this population [58]. Rural areas are also more susceptible to cultural stigmas often discouraging health-seeking behavior and disregarding chronic disease symptoms [59]. However, emerging evidence suggests that broader structural factors like economic policy, educational status and infrastructural capacity can play a critical role in health inequity. Hospitals in sparsely populated rural areas are notoriously underfunded and subject to hospital closures leading to reduced preventive care access and delayed treatment [60]. Studies have found detrimental economic effects of hospital closures on such communities but quality of care data is still pending. Funding formulas that adjust effectively for infrastructure deficits, transportation and specialized staff can be crucial to address mortality in these underserved populations [60]. These vulnerabilities of non-metropolitan areas were highlighted during the COVID-19 pandemic, with mortality rising steadily from 2018 to 2021 [61]. Rural hospitals were subject to closure with those available having limited access to personal protective equipment and ventilator machines [61]. Moreover, the virus was notoriously undertested in rural communities with governors often delaying statewide lockdown orders and physical distancing mandates, further exacerbating community spread [62–64].

From 1990 to 2019, the global burden of obesity and hypertension has seen a shift, with rising absolute burden now observed in higher sociodemographic index (SDI) regions of the world [65, 66]. Population aging, lifestyle changes (dietary sodium, obesity, inactivity) and improved detection/treatment have been accredited to shaping this shift. The US absolute burden bears marked similarities to these international trends but with distinct internal discrepancies. Unlike countries with centralized health coverage, the USA exhibits substantial geographic variability with mortality hotspots in rural, high-poverty areas and in states without Medicaid expansion. This pronounced heterogeneity in state-level infrastructure and policies present a unique challenge in establishing uniform structural interventions throughout the country. However, nations with universal healthcare systems also displayed rising absolute burdens, highlighting that healthcare access alone cannot fully counteract the influence of aging and lifestyle factors [65, 66]. Consequently, the rising obesity rates and state-level inequities could be critical factors in amplifying disease burden in the USA in comparison with other high-SDI countries. Understanding trends and disparities of hypertension and coexisting obesity signifies the importance of addressing both cardiovascular risk factors and policy reforms for adequate disease prevention.

### Public health implications

Globally, the incidence of obesity and hypertensive disease continues to rise to a pandemic level [26, 67]. Our results carry important clinical and public health implications. About 75% of individuals with hypertensive disease are overweight or obese; this overlapping risk underscores the importance of weight control in blood pressure management [68]. Moreover, an integrated management of these conditions could be cost efficient and productive. Clinicians should consider robust medical and surgical weight loss interventions in obese individuals with uncontrolled hypertensive disease. A multidisciplinary care team with a cardiologist, nutritionist and mental health professional should be considered. Improved disease outcomes in vulnerable communities require resident engagement and inclusivity in the context of municipal planning and policy development efforts. Local rather than national interventions are required to create a balanced food environment, ensuring equitable access in segregated communities. Furthermore, trial designs involving diverse demographics and balanced gender representation should be pushed to widen our evidence base. Male-focused outreach programs to improve and encourage routine blood pressure and cholesterol screening should be implemented, primarily focusing on high risk NH Black and rural populations [69]. At home BP monitoring and charting should be encouraged and taught. The possibility of using targeted advertisement to address gender-specific stigmas regarding mental health, obesity and seeking health care should be considered. Furthermore, the equitable enrollment of females in clinical trials should be made to widen our evidence base. For adolescents, proper education through workshops can aid school nurses in identifying early signs of metabolic syndrome and obesity [70]. To address racial inequities, weight loss regimens implemented for the NH Asian/Pacific Islander population should be tailored to the validated studies accounting for greater risk at lower BMIs. Notably, the reinforcement of preventive care models in emerging hotspot states like Rhode Island and Wyoming is crucial. State-level variations in AAMR are rooted in policy decisions (e.g., Medicaid expansion) and could be addressed through legislative action. Systemic flaws in infrastructure underinvestment and resource maldistribution can help mitigate the disproportionate rise in non-metropolitan areas of the USA. Moreover, careful consideration should be made into utilizing telemedicine when possible, expanding mobile clinics and improving hospital funding. In these underserved communities, implementing telemonitoring systems such as wearable heart monitors or blood pressure bands with Wi-Fi connectivity will allow healthcare providers to intervene early and adjust treatments when needed without requiring physical visits [71]. Moreover, tracking physical activity could ensure and encourage timely lifestyle modifications. Expanding telemedicine services will enable regular virtual consultations, prompt symptom management, timely medication adjustments leading to less hospital admissions and patient burden. Addressing the rising AAMRs in hypertensive disease and coexisting obesity requires a multipronged and culturally sensitive approach. Tailored interventions that address the disparities observed are essential to curb these variable trends and provide favorable and equitable cardiovascular health services. Ensuring that adequate efforts are made to address the rising rates of such chronic conditions will improve workforce productivity and have a positive impact on the US economy.

### Limitations

Our study has several limitations that should be acknowledged. Firstly, a key limitation lies in the reliance on death certificate data, where hypertensive disease and obesity may often be underreported or misclassified. These conditions frequently contribute to cardiovascular mortality rather than being listed as the primary underlying cause of death, leading to potential underestimation of their true impact. Moreover, the CDC WONDER database does not allow for clear differentiation between underlying and contributing causes when analyzing multiple conditions simultaneously, which may introduce ambiguity in attribution. Nonetheless, this approach has been used in previously published studies evaluating co-occurring conditions on death certificates as either underlying or contributing causes of death [72, 73]. Secondly, the dataset is limited to aggregate-level data and does not include individual-level clinical information, such as coexisting chronic illnesses (e.g., diabetes mellitus), lifestyle factors (e.g., tobacco use, physical activity), medication adherence or socioeconomic status. These unmeasured variables are known to influence both the incidence and outcomes of hypertensive disease and obesity, and their absence may introduce residual confounding into our analysis. As such, observed mortality trends may be partly attributable to these cofactors rather than the studied conditions alone. Thirdly, reporting of data could be impacted by differences in healthcare use, diagnostic perception and access to preventive care. Lastly, our retrospective use of secondary data sources like CDC WONDER, naturally restricts our capacity to prove causation or draw conclusions between treatments and observed mortality rates.

## Conclusion

Our study reveals a rising mortality due to hypertensive disease and coexisting obesity in the USA, with only a modest decline observed in the last three years. These trends reflect the complex association between metabolic health, socioeconomic factors and racial discrepancies in our healthcare system and associated cardiovascular risks involved. This dual epidemic of hypertensive disease and obesity requires a multifaceted approach which prioritizes timely screening, healthcare education, lifestyle modification and tailored treatment policies.

## Supplementary Information

Supplementary Material 1

## Data Availability

The data that support the findings of this study are openly available in CDC WONDER at [**https://wonder.cdc.gov/**](https:/wonder.cdc.gov).

## Abbreviations

NCDs: Non-communicable diseases
USA: United States
NH: Non-Hispanic
CDC WONDER: Center for Disease Control Wide-Ranging Online Data for Epidemiologic Research
ICD-10: International Classification of Diseases, Tenth Revision
STROBE: Strengthening the Reporting of Observational Studies in Epidemiology
IRB: Institutional review board
CMR: Crude mortality rates
AAMRs: Age-adjusted mortality rates
APC: Annual percentage change
AAPC: Average annual percentage change
ACA: Affordable Care Act
