# Supplementary material for: Trends and disparities in mortality due to hypertensive disease and coexisting obesity in the USA: 1999–2023
Source: Egypt Heart J. 2025 Aug 26;77:81. doi: 10.1186/s43044-025-00677-5 (PMC12380657; doi:10.1186/s43044-025-00677-5)
Supplement: Supplementary file 1 — Supplementary Material 1 [file 43044_2025_677_MOESM1_ESM.docx]

**Supplemental Table 1** Obesity and Hypertension–related Mortality, Stratified by Sex and Race in Adults in the United States, 1999 to 2023.

| **Deaths** | | | | | | | | | |
| --- | --- | --- | --- | --- | --- | --- | --- | --- | --- |
| **Year** | **Overall** | **Women** | **Men** | **NH White** | **NH Black or African American** | **NH Asian or Pacific Islander** | **NH American Indian or Alaska Native** | **Hispanic or Latino** | **Population** |
| 1999 | 2351 | 1313 | 1038 | 1589 | 612 | 15 | 10 | 114 | 180408769 |
| 2000 | 4645 | 2471 | 2174 | 3283 | 1070 | 29 | 27 | 216 | 181984640 |
| 2001 | 4835 | 2538 | 2297 | 3374 | 1152 | 18 | 31 | 242 | 184305128 |
| 2002 | 5741 | 2970 | 2771 | 4006 | 1359 | 34 | 38 | 282 | 186208028 |
| 2003 | 6587 | 3390 | 3197 | 4593 | 1511 | 43 | 46 | 370 | 188090429 |
| 2004 | 7144 | 3579 | 3565 | 4917 | 1715 | 47 | 47 | 394 | 190205384 |
| 2005 | 7906 | 3928 | 3978 | 5584 | 1771 | 72 | 63 | 401 | 192551384 |
| 2006 | 8412 | 4121 | 4291 | 5843 | 1858 | 64 | 88 | 532 | 195019359 |
| 2007 | 9146 | 4363 | 4783 | 6378 | 1984 | 95 | 73 | 598 | 197403777 |
| 2008 | 9988 | 4767 | 5221 | 6996 | 2132 | 120 | 82 | 622 | 199795090 |
| 2009 | 11168 | 5172 | 5996 | 7674 | 2388 | 89 | 136 | 814 | 202107016 |
| 2010 | 11908 | 5450 | 6458 | 8234 | 2521 | 123 | 132 | 844 | 203891983 |
| 2011 | 13295 | 6083 | 7212 | 9291 | 2712 | 141 | 125 | 973 | 206592936 |
| 2012 | 14690 | 6662 | 8028 | 10247 | 3007 | 140 | 159 | 1064 | 208826037 |
| 2013 | 15687 | 7074 | 8613 | 10891 | 3202 | 133 | 167 | 1225 | 211085314 |
| 2014 | 17131 | 7561 | 9570 | 11882 | 3434 | 177 | 201 | 1343 | 213809280 |
| 2015 | 18645 | 8167 | 10478 | 12845 | 3828 | 228 | 180 | 1447 | 216553817 |
| 2016 | 19798 | 8714 | 11084 | 13594 | 4073 | 199 | 226 | 1592 | 218641417 |
| 2017 | 20911 | 8963 | 11948 | 14477 | 4142 | 241 | 223 | 1705 | 221447331 |
| 2018 | 22441 | 9433 | 13008 | 15586 | 4448 | 261 | 253 | 1785 | 223311190 |
| 2019 | 24038 | 10099 | 13939 | 16587 | 4796 | 292 | 284 | 1986 | 224981167 |
| 2020 | 37307 | 16175 | 21132 | 23464 | 8152 | 509 | 506 | 4572 | 226635013 |
| 2021 | 45410 | 19656 | 25754 | 29808 | 9375 | 474 | 531 | 4805 | 228238412 |
| 2022 | 38758 | 16830 | 21928 | 26535 | 7584 | 408 | 435 | 3373 | 229508599 |
| 2023 | 34825 | 14899 | 19926 | 23820 | 6843 | 395 | 384 | 2951 | 231529762 |
| **Total** | 412767 | 184378 | 228389 | 281498 | 85669 | 4347 | 4447 | 34250 | 5163131262 |

NH, non-Hispanic.

**Supplemental Table 2.** Obesity and Hypertension–related Mortality, Stratified by Place of Death in Adults in the United States, 1999 to 2023

| **Deaths** | | | | |
| --- | --- | --- | --- | --- |
| **Year** | **Medical**  **Facility** | **Nursing Home/Long-term**  **Care Facility** | **Hospices** | **Home** |
| 1999 | 1294 | 148 | Missing | 833 |
| 2000 | 2492 | 296 | Missing | 1684 |
| 2001 | 2601 | 329 | Missing | 1695 |
| 2002 | 2995 | 370 | Missing | 2144 |
| 2003 | 3362 | 423 | 14 | 2470 |
| 2004 | 3660 | 433 | 25 | 2697 |
| 2005 | 3964 | 522 | 45 | 3038 |
| 2006 | 4029 | 561 | 50 | 3397 |
| 2007 | 4405 | 518 | 62 | 3686 |
| 2008 | 4639 | 562 | 54 | 4091 |
| 2009 | 5053 | 599 | 94 | 4614 |
| 2010 | 5401 | 671 | 130 | 5101 |
| 2011 | 5836 | 794 | 160 | 5832 |
| 2012 | 6322 | 908 | 166 | 6514 |
| 2013 | 6506 | 865 | 187 | 7228 |
| 2014 | 6940 | 1067 | 240 | 7937 |
| 2015 | 7455 | 1040 | 294 | 8820 |
| 2016 | 7636 | 1091 | 330 | 9537 |
| 2017 | 7895 | 1117 | 372 | 10259 |
| 2018 | 8448 | 1193 | 529 | 11000 |
| 2019 | 8714 | 1279 | 675 | 11988 |
| 2020 | 15296 | 1854 | 700 | 17383 |
| 2021 | 19343 | 1855 | 570 | 20659 |
| 2022 | 14114 | 1908 | Missing | 19205 |
| 2023 | 11940 | 1924 | Missing | 17756 |
| **Total** | 170340 | 22327 | 4697 | 189568 |

**Supplemental Table 3** Annual percent change (APC) of Obesity and Hypertension–related Age-Adjusted Mortality Rates per 100,000 in Adults in the United States, 1999 to 2023

| **Year Interval** | **APC (95% CI)** | **P value** |
| --- | --- | --- |
| **Overall** | | |
| 1999-2001 | 39.65* (8.16-79.65) | < 0.001 |
| 2001-2018 | 6.65 (-0.39-7.46) | >0.05 |
| 2018-2021 | 23.96* (16.86-28.38) | < 0.001 |
| 2021-2023 | -13.24* (-20.02--6.27) | < 0.001 |
| **Men** | | |
| 1999-2001 | 45.53* (10.15-87.60) | < 0.001 |
| 2001-2018 | 7.78* (2.84-8.60) | < 0.001 |
| 2018-2021 | 22.87* (16.40-26.94) | < 0.001 |
| 2021-2023 | -12.72* (-19.19--6.27) | < 0.001 |
| **Women** | | |
| 1999-2001 | 35.32* (7.01-68.65) | < 0.001 |
| 2001-2018 | 5.53 (-1.63-6.31) | >0.05 |
| 2018-2021 | 24.79* (17.13-29.61) | < 0.001 |
| 2021-2023 | -14.11* (-21.26--6.87) | < 0.001 |
| **Young Adults (25-44 years)** | | |
| 1999-2001 | 44.38* (23.97-64.13) | < 0.001 |
| 2001-2015 | 8.42* (7.79-9.51) | < 0.001 |
| 2015-2018 | 1.44 (-1.62-5.35) | >0.05 |
| 2018-2021 | 23.06* (18.68-27.08) | < 0.001 |
| 2021-2023 | -15.03* (-19.64--10.76 | < 0.001 |
| **Middle Aged Adults (45-64 years)** | | |
| 1999-2001: | 43.08* (8.59-82.74) | < 0.001 |
| 2001-2018 | 6.36* (1.85-7.13) | < 0.001 |
| 2018-2021 | 22.59* (15.66-26.63) | < 0.001 |
| 2021-2023 | -15.27* (-22.01--8.70 | < 0.001 |
| **Older Adults (65 years and above)** | | |
| 1999-2018 | 7.24* (6.02-8.34) | < 0.001 |
| 2018-2021 | 27.30* (19.85-31.78) | < 0.001 |
| 2021-2023 | -11.18* (-17.94--3.89) | < 0.001 |
| **NH American Indian or Alaska Native** | | |
| 1999-2018 | 8.82* (5.93-10.80) | < 0.001 |
| 2018-2021 | 28.53* (17.09-36.67) | < 0.001 |
| 2021-2023 | -15.74* (-25.83--3.18) | < 0.001 |
| **NH Black or African American** | | |
| 1999-2001 | 34.57* (6.59-67.92) | < 0.001 |
| 2001-2018 | 5.11 (-1.52-5.97) | >0.05 |
| 2018-2021 | 27.27* (19.40-32.26) | < 0.001 |
| 2021-2023 | -16.69* (-23.63--10.01) | < 0.001 |
| **Hispanic or Latino** | | |
| 1999-2018 | 7.17* (4.57-9.39) | < 0.001 |
| 2018-2021 | 33.99* (20.44-43.76) | < 0.001 |
| 2021-2023 | -29.06* (-38.33--17.97) | < 0.001 |
| **NH Asian or Pacific Islander** | | |
| 1999-2018 | 7.00 (-1.54-11.37) | >0.05 |
| 2018-2021 | 21.19* (9.64-29.72) | < 0.001 |
| 2021-2023 | -15.76* (-25.65--1.00) | < 0.001 |
| **NH White** | | |
| 1999-2001 | 40.53* (9.94-72.05) | < 0.001 |
| 2001-2018 | 7.30* (4.77-8.01) | < 0.001 |
| 2018-2021 | 21.87* (15.85-25.65 | < 0.001 |
| **Non-metropolitan areas** | | |
| 1999-2001 | 45.74* (16.70-72.01) | < 0.001 |
| 2001-2018 | 7.49* (6.20-8.17) | < 0.001 |
| 2018-2020 | 23.59* (13.86-29.29) | < 0.001 |
| **Metropolitan area** | | |
| 1999-2001 | 37.12* (8.17-74.72) | < 0.001 |
| 2001-2018 | 6.66 (-1.07-7.47) | >0.05 |
| 2018-2020 | 22.93* (10.32-30.65) | < 0.001 |
| **Northeast** | | |
| 1999-2011 | 9.94* (8.55-15.86) | < 0.001 |
| 2011-2018 | 4.85 (-2.09-6.96) | >0.05 |
| 2018-2021 | 17.81* (11.68-22.87) | < 0.001 |
| 2021-2023 | -11.30* (-17.36--3.90) | < 0.001 |
| **Midwest** | | |
| 1999-2018 | 8.03* (6.37-9.11) | < 0.001 |
| 2018-2021 | 21.12* (13.24-25.48 | < 0.001 |
| 2021-2023 | -15.46* (-22.99--5.58) | < 0.001 |
| **South** | | |
| 1999-2001 | 42.52* (8.40-84.12) | < 0.001 |
| 2001-2018 | 6.71* (1.37-7.57) | < 0.001 |
| 2018-2021 | 29.17* (22.47-33.49) | < 0.001 |
| 2021-2023 | -13.42* (-19.67--7.64) | < 0.001 |
| **West** | | |
| 1999-2002 | 26.10* (9.15-71.12) | < 0.001 |
| 2002-2018 | 5.11* (2.13-5.91) | < 0.001 |
| 2018-2021 | 22.67* (15.72-26.64) | < 0.001 |
| 2021-2023 | -13.24* (-19.92--6.23) | < 0.001 |

APC = Annual percent change; NH = non-Hispanic. N/A = unreliable or suppressed

**Supplemental Table 4** Overall and Sex‐Stratified Obesity and Hypertension–related Age-Adjusted Mortality Rates per 100,000 in Adults in the United States from 1999 to 2023

| **Age-Adjusted Rate (95% CI)** | | | |
| --- | --- | --- | --- |
| **Year** | **Men** | **Women** | **Overall** |
| 1999 | 1.25 (1.17–1.33) | 1.35 (1.28–1.43) | 1.32 (1.26–1.37) |
| 2000 | 2.58 (2.47–2.69) | 2.53 (2.43–2.63) | 2.58 (2.50–2.65) |
| 2001 | 2.67 (2.56–2.77) | 2.55 (2.45–2.65) | 2.64 (2.57–2.72) |
| 2002 | 3.16 (3.04–3.28) | 2.93 (2.82–3.03) | 3.07 (2.99–3.15) |
| 2003 | 3.54 (3.42–3.67) | 3.31 (3.19–3.42) | 3.44 (3.36–3.52) |
| 2004 | 3.87 (3.74–3.99) | 3.44 (3.33–3.56) | 3.68 (3.60–3.77) |
| 2005 | 4.21 (4.08–4.35) | 3.69 (3.57–3.80) | 3.98 (3.89–4.07) |
| 2006 | 4.45 (4.32–4.59) | 3.79 (3.68–3.91) | 4.15 (4.06–4.24) |
| 2007 | 4.89 (4.75–5.03) | 3.97 (3.85–4.09) | 4.44 (4.34–4.53) |
| 2008 | 5.24 (5.09–5.38) | 4.24 (4.12–4.37) | 4.74 (4.65–4.84) |
| 2009 | 5.86 (5.71–6.01) | 4.57 (4.45–4.70) | 5.18 (5.08–5.28) |
| 2010 | 6.26 (6.11–6.42) | 4.73 (4.60–4.85) | 5.47 (5.37–5.57) |
| 2011 | 6.88 (6.72–7.04) | 5.16 (5.03–5.29) | 6.02 (5.91–6.12) |
| 2012 | 7.50 (7.33–7.67) | 5.58 (5.44–5.72) | 6.55 (6.44–6.66) |
| 2013 | 7.93 (7.76–8.10) | 5.85 (5.71–5.99) | 6.86 (6.75–6.97) |
| 2014 | 8.72 (8.54–8.90) | 6.10 (5.96–6.24) | 7.39 (7.27–7.50) |
| 2015 | 9.31 (9.13–9.50) | 6.52 (6.37–6.66) | 7.89 (7.77–8.01) |
| 2016 | 9.78 (9.59–9.96) | 6.85 (6.70–7.00) | 8.28 (8.16–8.40) |
| 2017 | 10.33 (10.14–10.52) | 6.92 (6.77–7.06) | 8.57 (8.45–8.69) |
| 2018 | 11.10 (10.91–11.30) | 7.17 (7.02–7.32) | 9.07 (8.95–9.19) |
| 2019 | 11.78 (11.58–11.98) | 7.56 (7.41–7.71) | 9.62 (9.49–9.74) |
| 2020 | 17.64 (17.40–17.89) | 11.85 (11.66–12.04) | 14.69 (14.54–14.85) |
| 2021 | 21.13 (20.87–21.40) | 14.49 (14.28–14.70) | 17.77 (17.61–17.94) |
| 2022 | 17.77 (17.53–18.01) | 12.15 (11.96–12.34) | 14.90 (14.75–15.05) |
| 2023 | 16.05 (15.82–16.28) | 10.56 (10.38–10.73) | 13.23 (13.09–13.37) |
| **Overall** | 8.16 (7.99-8.32) | 5.91 (5.78-6.05) | 7.02 (6.91-7.12) |

**Supplemental Table 5** Obesity and Hypertension –related Mortality, Stratified by Age group in Adults in the United States, 1999 to 2023.

|  | **Deaths** | | | **Age-Adjusted Rate (95% CI)** | | |
| --- | --- | --- | --- | --- | --- | --- |
| **Year** | **Young Adults** | **Middle Aged Adults** | **Older Adults** | **Young Adults** | **Middle Aged Adults** | **Older Adults** |
| 1999 | 378 | 1087 | 886 | 0.42 (0.38–0.46) | 1.81 (1.71–1.92) | 2.56 (2.39–2.73) |
| 2000 | 685 | 2147 | 1813 | 0.79 (0.73–0.85) | 3.49 (3.34–3.64) | 5.19 (4.95–5.43) |
| 2001 | 729 | 2388 | 1718 | 0.89 (0.83–0.96) | 3.75 (3.60–3.90) | 4.84 (4.61–5.06) |
| 2002 | 866 | 2800 | 2075 | 1.05 (0.98–1.11) | 4.21 (4.05–4.36) | 5.84 (5.58–6.09) |
| 2003 | 951 | 3367 | 2269 | 1.15 (1.08–1.23) | 4.86 (4.70–5.03) | 6.34 (6.08–6.60) |
| 2004 | 1015 | 3698 | 2431 | 1.25 (1.18–1.33) | 5.20 (5.04–5.37) | 6.73 (6.46–7.00) |
| 2005 | 1109 | 4083 | 2714 | 1.35 (1.27–1.43) | 5.56 (5.39–5.73) | 7.41 (7.13–7.69) |
| 2006 | 1176 | 4366 | 2870 | 1.46 (1.38–1.55) | 5.72 (5.55–5.89) | 7.73 (7.45–8.01) |
| 2007 | 1321 | 4814 | 3011 | 1.67 (1.58–1.76) | 6.14 (5.97–6.31) | 7.96 (7.68–8.25) |
| 2008 | 1394 | 5241 | 3353 | 1.73 (1.64–1.82) | 6.56 (6.38–6.74) | 8.66 (8.37–8.95) |
| 2009 | 1554 | 6050 | 3564 | 1.94 (1.84–2.03) | 7.36 (7.17–7.54) | 9.01 (8.72–9.31) |
| 2010 | 1624 | 6246 | 4038 | 2.05 (1.95–2.15) | 7.46 (7.27–7.64) | 10.04 (9.73–10.35) |
| 2011 | 1774 | 7028 | 4493 | 2.25 (2.15–2.36) | 8.30 (8.10–8.49) | 10.89 (10.57–11.21) |
| 2012 | 1921 | 7604 | 5165 | 2.46 (2.35–2.57) | 8.93 (8.73–9.14) | 12.00 (11.67–12.33) |
| 2013 | 1970 | 8026 | 5691 | 2.53 (2.41–2.64) | 9.33 (9.13–9.54) | 12.74 (12.40–13.07) |
| 2014 | 2181 | 8585 | 6365 | 2.78 (2.66–2.90) | 9.93 (9.72–10.15) | 13.78 (13.44–14.12) |
| 2015 | 2309 | 9249 | 7087 | 2.94 (2.82–3.06) | 10.59 (10.37–10.81) | 14.83 (14.49–15.18) |
| 2016 | 2460 | 9814 | 7524 | 3.15 (3.03–3.28) | 11.19 (10.96–11.42) | 15.25 (14.90–15.60) |
| 2017 | 2540 | 10228 | 8143 | 3.15 (3.03–3.28) | 11.63 (11.39–11.86) | 15.98 (15.63–16.33) |
| 2018 | 2661 | 10651 | 9129 | 3.31 (3.18–3.44) | 12.06 (11.83–12.30) | 17.41 (17.05–17.77) |
| 2019 | 2807 | 11228 | 10003 | 3.46 (3.33–3.59) | 12.80 (12.55–13.04) | 18.55 (18.18–18.92) |
| 2020 | 4114 | 16663 | 16530 | 5.04 (4.88–5.19) | 19.12 (18.82–19.42) | 29.71 (29.25–30.17) |
| 2021 | 4983 | 20417 | 20010 | 5.99 (5.82–6.16) | 23.29 (22.96–23.62) | 35.89 (35.38–36.39) |
| 2022 | 4119 | 16202 | 18437 | 4.88 (4.73–5.03) | 18.62 (18.32–18.91) | 32.01 (31.54–32.48) |
| 2023 | 3727 | 14409 | 16689 | 4.35 (4.21–4.49) | 16.56 (16.29–16.84) | 28.31 (27.88–28.75) |
| **Overall** | 50368 | 196391 | 166008 | 1.81 (1.71-1.92) | 2.56 (2.39-2.73) | 13.59 (13.26-13.91) |

Young Adult = 25-44 years; Middle Aged Adults = 45-64 years; Older Adults = 65 years and above

**Supplemental Table 6** Obesity and Hypertension–related Age-Adjusted Mortality Rates per 100,000 stratified by Race in Adults in the United States from 1999 to 2023

| **Age-Adjusted Rate (95% CI)** | | | | | |
| --- | --- | --- | --- | --- | --- |
| **Year** | **NH White** | **NH Black or African American** | **NH American Indian or Alaska Native** | **Hispanic or Latino** | **NH Asian or Pacific Islander** |
| 1999 | 1.11 (1.05–1.16) | 3.43 (3.15–3.70) | Unreliable (0.48–2.20) | 0.94 (0.75–1.12) | Unreliable (0.13–0.39) |
| 2000 | 2.30 (2.22–2.37) | 5.94 (5.58–6.31) | 2.72 (1.74–4.05) | 1.63 (1.40–1.87) | 0.46 (0.30–0.67) |
| 2001 | 2.34 (2.26–2.42) | 6.20 (5.84–6.57) | 2.59 (1.73–3.72) | 1.77 (1.53–2.00) | Unreliable (0.16–0.46) |
| 2002 | 2.73 (2.65–2.82) | 7.11 (6.72–7.49) | 3.50 (2.41–4.92) | 1.95 (1.71–2.19) | 0.50 (0.34–0.71) |
| 2003 | 3.09 (3.00–3.18) | 7.73 (7.33–8.13) | 4.03 (2.88–5.49) | 2.53 (2.25–2.80) | 0.61 (0.44–0.83) |
| 2004 | 3.27 (3.18–3.36) | 8.57 (8.16–8.98) | 3.76 (2.70–5.10) | 2.41 (2.16–2.66) | 0.61 (0.44–0.81) |
| 2005 | 3.66 (3.56–3.76) | 8.68 (8.27–9.09) | 5.02 (3.80–6.51) | 2.36 (2.11–2.61) | 0.93 (0.72–1.18) |
| 2006 | 3.79 (3.69–3.89) | 8.81 (8.40–9.22) | 6.56 (5.20–8.17) | 2.96 (2.69–3.23) | 0.74 (0.57–0.96) |
| 2007 | 4.07 (3.97–4.17) | 9.18 (8.76–9.59) | 5.16 (3.99–6.57) | 3.07 (2.81–3.33) | 1.09 (0.88–1.34) |
| 2008 | 4.41 (4.30–4.51) | 9.59 (9.18–10.01) | 5.82 (4.58–7.30) | 3.17 (2.91–3.44) | 1.32 (1.08–1.56) |
| 2009 | 4.78 (4.67–4.89) | 10.56 (10.13–11.00) | 9.92 (8.16–11.67) | 3.92 (3.63–4.20) | 0.88 (0.71–1.09) |
| 2010 | 5.10 (4.99–5.21) | 10.85 (10.42–11.29) | 8.75 (7.20–10.29) | 3.99 (3.70–4.27) | 1.27 (1.04–1.50) |
| 2011 | 5.70 (5.58–5.82) | 11.44 (11.00–11.88) | 8.09 (6.62–9.57) | 4.35 (4.06–4.64) | 1.36 (1.13–1.59) |
| 2012 | 6.21 (6.09–6.34) | 12.47 (12.01–12.92) | 10.17 (8.53–11.81) | 4.52 (4.24–4.81) | 1.32 (1.09–1.54) |
| 2013 | 6.54 (6.41–6.67) | 13.09 (12.63–13.56) | 10.50 (8.86–12.14) | 5.07 (4.77–5.36) | 1.19 (0.98–1.39) |
| 2014 | 7.10 (6.96–7.23) | 13.59 (13.12–14.05) | 12.85 (11.01–14.69) | 5.18 (4.90–5.47) | 1.49 (1.27–1.72) |
| 2015 | 7.55 (7.42–7.69) | 14.80 (14.32–15.28) | 10.75 (9.14–12.37) | 5.38 (5.09–5.67) | 1.89 (1.64–2.14) |
| 2016 | 7.96 (7.82–8.10) | 15.39 (14.91–15.87) | 13.89 (12.03–15.75) | 5.75 (5.46–6.04) | 1.55 (1.33–1.77) |
| 2017 | 8.35 (8.20–8.49) | 15.36 (14.88–15.84) | 12.92 (11.18–14.67) | 5.84 (5.55–6.13) | 1.82 (1.59–2.06) |
| 2018 | 8.89 (8.74–9.03) | 16.28 (15.79–16.77) | 14.72 (12.86–16.59) | 5.90 (5.62–6.19) | 1.88 (1.65–2.12) |
| 2019 | 9.34 (9.19–9.49) | 17.27 (16.77–17.77) | 16.30 (14.35–18.25) | 6.33 (6.05–6.62) | 2.04 (1.80–2.27) |
| 2020 | 13.10 (12.92–13.28) | 28.78 (28.14–29.43) | 28.21 (25.68–30.74) | 14.59 (14.15–15.03) | 3.44 (3.13–3.74) |
| 2021 | 16.78 (16.57–16.98) | 33.60 (32.91–34.30) | 31.97 (29.16–34.78) | 14.65 (14.22–15.08) | 3.30 (3.00–3.60) |
| 2022 | 14.74 (14.55–14.93) | 27.02 (26.40–27.65) | 27.11 (24.50–29.73) | 10.03 (9.68–10.37) | 2.72 (2.46–2.99) |
| 2023 | 13.18 (13.01–13.36) | 23.87 (23.29–24.44) | 23.47 (21.06–25.88) | 8.36 (8.05–8.67) | 2.59 (2.33–2.85) |
| **Overall** | 6.64 (6.52-6.77) | 13.58 (13.1244-14.04) | 11.61 (9.59-12.92) | 5.06 (4.78-5.35) | 1.52 (1.21-1.65) |

NH=non-Hispanic

**Supplemental Table 7A** Obesity and Hypertension–related Age-Adjusted Mortality Rates per 100,000 Stratified by State in Adults in the United States, 1999 to 2020.

| **State** | **Age-Adjusted Rate (95% CI)** |
| --- | --- |
| Connecticut | 3.23 (3.08–3.37) |
| Alabama | 3.35 (3.22–3.48) |
| Virginia | 3.45 (3.34–3.55) |
| Massachusetts | 3.54 (3.43–3.65) |
| Missouri | 4.44 (4.31–4.58) |
| Nebraska | 4.56 (4.31–4.81) |
| Maine | 4.61 (4.33–4.89) |
| New Jersey | 4.66 (4.55–4.77) |
| Illinois | 4.71 (4.61–4.81) |
| Utah | 4.90 (4.65–5.14) |
| New Hampshire | 4.90 (4.61–5.20) |
| Pennsylvania | 4.96 (4.87–5.06) |
| Hawaii | 5.12 (4.81–5.43) |
| Kansas | 5.13 (4.92–5.35) |
| South Dakota | 5.20 (4.81–5.60) |
| Arizona | 5.30 (5.15–5.44) |
| Arkansas | 5.37 (5.15–5.59) |
| Florida | 5.38 (5.30–5.46) |
| Idaho | 5.57 (5.26–5.88) |
| Washington | 5.68 (5.54–5.83) |
| Maryland | 5.83 (5.67–5.99) |
| Indiana | 5.99 (5.83–6.14) |
| Oregon | 6.17 (5.98–6.37) |
| Georgia | 6.37 (6.24–6.51) |
| New York | 6.43 (6.33–6.52) |
| Michigan | 6.43 (6.30–6.55) |
| California | 6.46 (6.39–6.52) |
| South Carolina | 6.48 (6.29–6.66) |
| Montana | 6.51 (6.11–6.90) |
| Nevada | 6.59 (6.34–6.85) |
| Kentucky | 6.66 (6.46–6.86) |
| Ohio | 6.72 (6.60–6.84) |
| New Mexico | 6.74 (6.45–7.04) |
| North Carolina | 6.87 (6.73–7.00) |
| North Dakota | 7.02 (6.51–7.52) |
| Minnesota | 7.12 (6.94–7.31) |
| Iowa | 7.17 (6.93–7.40) |
| Alaska | 7.25 (6.65–7.85) |
| Louisiana | 7.48 (7.27–7.69) |
| Colorado | 7.65 (7.45–7.85) |
| Tennessee | 7.78 (7.61–7.96) |
| Texas | 7.82 (7.73–7.92) |
| Wyoming | 7.91 (7.31–8.52) |
| Wisconsin | 8.16 (7.98–8.35) |
| Rhode Island | 8.26 (7.83–8.70) |
| West Virginia | 8.56 (8.22–8.89) |
| Delaware | 8.56 (8.08–9.05) |
| Mississippi | 8.59 (8.31–8.86) |
| District of Columbia | 11.63 (10.91–12.36) |
| Oklahoma | 13.16 (12.86–13.47) |
| Vermont | 15.11 (14.37–15.85) |
| **Overall** | 6.54 (6.29-6.79) |

**Supplemental Table 7B** Obesity and Hypertension–related Age-Adjusted Mortality Rates per 100,000 Stratified by State in Adults in the United States, 2021 to 2023.

| **State** | **Age-Adjusted Rate (95% CI)** |
| --- | --- |
| Massachusetts | 7.56 (7.12–7.99) |
| Virginia | 7.69 (7.30–8.08) |
| New Jersey | 7.75 (7.37–8.12) |
| Connecticut | 8.75 (8.10–9.40) |
| Hawaii | 9.39 (8.31–10.47) |
| Maine | 9.80 (8.75–10.86) |
| Illinois | 10.94 (10.55–11.33) |
| Missouri | 11.11 (10.55–11.68) |
| New Hampshire | 11.14 (10.01–12.26) |
| California | 11.33 (11.11–11.56) |
| Alabama | 11.68 (11.04–12.32) |
| Florida | 12.12 (11.82–12.42) |
| New York | 12.18 (11.86–12.51) |
| North Carolina | 12.40 (11.95–12.85) |
| Nebraska | 12.66 (11.59–13.73) |
| Pennsylvania | 13.42 (13.01–13.84) |
| South Dakota | 13.77 (12.13–15.40) |
| Michigan | 13.95 (13.46–14.44) |
| Ohio | 13.97 (13.51–14.42) |
| Utah | 14.46 (13.50–15.43) |
| Indiana | 14.54 (13.93–15.16) |
| Washington | 14.93 (14.36–15.51) |
| Kentucky | 15.10 (14.34–15.87) |
| Oregon | 15.18 (14.42–15.94) |
| Kansas | 15.70 (14.71–16.68) |
| Arizona | 15.72 (15.10–16.34) |
| Maryland | 16.03 (15.36–16.71) |
| Idaho | 16.51 (15.29–17.72) |
| Arkansas | 16.52 (15.53–17.52) |
| New Mexico | 16.62 (15.43–17.80) |
| Alaska | 16.87 (14.74–19.00) |
| Montana | 18.17 (16.50–19.84) |
| Minnesota | 18.40 (17.65–19.14) |
| Nevada | 18.82 (17.81–19.84) |
| Texas | 18.92 (18.57–19.27) |
| Tennessee | 19.80 (19.08–20.51) |
| Mississippi | 20.23 (19.10–21.36) |
| Colorado | 20.39 (19.60–21.18) |
| North Dakota | 20.42 (18.18–22.66) |
| Iowa | 20.95 (19.88–22.01) |
| Georgia | 21.79 (21.19–22.40) |
| Louisiana | 23.48 (22.51–24.45) |
| Wisconsin | 23.90 (23.08–24.72) |
| West Virginia | 24.60 (23.02–26.18) |
| District of Columbia | 25.67 (22.91–28.43) |
| Vermont | 26.01 (23.54–28.48) |
| Wyoming | 27.14 (24.33–29.95) |
| Rhode Island | 29.86 (27.84–31.89) |
| Delaware | 34.25 (31.95–36.54) |
| South Carolina | 41.36 (40.22–42.50) |
| Oklahoma | 47.42 (45.95–48.88) |
| **Overall** | 17.67 (16.65-18.70) |

**Supplemental Table 8.** Obesity and Hypertension–related Age-Adjusted Mortality Rate per 100,000 Stratified by Census Region in Adults in the United States 1999-2023.

| **Census Region** | **Year** | **Age-Adjusted Rate (95% CI)** |
| --- | --- | --- |
| Northeast | 1999 | 1.16 (1.05–1.27) |
| Northeast | 2000 | 2.24 (2.09–2.40) |
| Northeast | 2001 | 2.27 (2.12–2.43) |
| Northeast | 2002 | 2.55 (2.39–2.71) |
| Northeast | 2003 | 2.75 (2.59–2.92) |
| Northeast | 2004 | 3.06 (2.89–3.24) |
| Northeast | 2005 | 3.24 (3.06–3.42) |
| Northeast | 2006 | 3.41 (3.23–3.59) |
| Northeast | 2007 | 3.98 (3.78–4.18) |
| Northeast | 2008 | 4.24 (4.03–4.44) |
| Northeast | 2009 | 4.75 (4.53–4.96) |
| Northeast | 2010 | 5.20 (4.98–5.42) |
| Northeast | 2011 | 5.52 (5.29–5.75) |
| Northeast | 2012 | 5.99 (5.76–6.23) |
| Northeast | 2013 | 6.10 (5.86–6.34) |
| Northeast | 2014 | 6.50 (6.26–6.75) |
| Northeast | 2015 | 7.14 (6.88–7.40) |
| Northeast | 2016 | 7.41 (7.15–7.67) |
| Northeast | 2017 | 7.60 (7.33–7.86) |
| Northeast | 2018 | 7.94 (7.67–8.21) |
| Northeast | 2019 | 8.28 (8.00–8.55) |
| Northeast | 2020 | 12.19 (11.86–12.53) |
| Northeast | 2021 | 12.41 (12.08–12.74) |
| Northeast | 2022 | 11.64 (11.32–11.96) |
| Northeast | 2023 | 10.14 (9.84–10.44) |
| Northeast | **Overall** | 5.90 (5.68-6.14) |
| Midwest | 1999 | 1.26 (1.15–1.37) |
| Midwest | 2000 | 2.50 (2.35–2.66) |
| Midwest | 2001 | 2.52 (2.37–2.67) |
| Midwest | 2002 | 2.89 (2.73–3.06) |
| Midwest | 2003 | 3.16 (2.99–3.33) |
| Midwest | 2004 | 3.37 (3.20–3.54) |
| Midwest | 2005 | 3.85 (3.67–4.03) |
| Midwest | 2006 | 3.82 (3.64–4.00) |
| Midwest | 2007 | 3.97 (3.79–4.15) |
| Midwest | 2008 | 4.61 (4.42–4.81) |
| Midwest | 2009 | 4.70 (4.51–4.90) |
| Midwest | 2010 | 5.15 (4.95–5.36) |
| Midwest | 2011 | 5.96 (5.74–6.18) |
| Midwest | 2012 | 6.38 (6.15–6.61) |
| Midwest | 2013 | 7.00 (6.76–7.23) |
| Midwest | 2014 | 7.78 (7.53–8.03) |
| Midwest | 2015 | 8.33 (8.07–8.58) |
| Midwest | 2016 | 8.56 (8.30–8.82) |
| Midwest | 2017 | 8.78 (8.52–9.04) |
| Midwest | 2018 | 9.45 (9.17–9.72) |
| Midwest | 2019 | 9.85 (9.58–10.13) |
| Midwest | 2020 | 15.03 (14.70–15.37) |
| Midwest | 2021 | 17.63 (17.26–17.99) |
| Midwest | 2022 | 14.47 (14.14–14.80) |
| Midwest | 2023 | 12.54 (12.23–12.85) |
| Midwest | **Overall** | 6.94 (6.72-7.17) |
| South | 1999 | 1.27 (1.18–1.36) |
| South | 2000 | 2.70 (2.58–2.83) |
| South | 2001 | 2.82 (2.69–2.95) |
| South | 2002 | 3.13 (3.00–3.27) |
| South | 2003 | 3.67 (3.53–3.81) |
| South | 2004 | 3.90 (3.75–4.04) |
| South | 2005 | 4.14 (3.99–4.28) |
| South | 2006 | 4.44 (4.28–4.59) |
| South | 2007 | 4.56 (4.41–4.72) |
| South | 2008 | 4.85 (4.69–5.00) |
| South | 2009 | 5.58 (5.41–5.75) |
| South | 2010 | 5.69 (5.52–5.86) |
| South | 2011 | 6.10 (5.93–6.28) |
| South | 2012 | 6.79 (6.61–6.97) |
| South | 2013 | 7.23 (7.05–7.42) |
| South | 2014 | 7.79 (7.60–7.98) |
| South | 2015 | 8.22 (8.02–8.41) |
| South | 2016 | 8.71 (8.51–8.91) |
| South | 2017 | 9.10 (8.90–9.30) |
| South | 2018 | 9.63 (9.42–9.83) |
| South | 2019 | 10.54 (10.32–10.75) |
| South | 2020 | 16.46 (16.20–16.73) |
| South | 2021 | 21.27 (20.97–21.57) |
| South | 2022 | 17.54 (17.27–17.81) |
| South | 2023 | 15.79 (15.54–16.04) |
| South | **Overall** | 7.68 (7.49-7.86) |
| West | 1999 | 1.70 (1.56–1.83) |
| West | 2000 | 2.84 (2.67–3.01) |
| West | 2001 | 2.81 (2.65–2.98) |
| West | 2002 | 3.64 (3.45–3.83) |
| West | 2003 | 4.02 (3.82–4.21) |
| West | 2004 | 4.12 (3.92–4.31) |
| West | 2005 | 4.47 (4.27–4.67) |
| West | 2006 | 4.66 (4.46–4.86) |
| West | 2007 | 5.06 (4.85–5.27) |
| West | 2008 | 5.24 (5.03–5.45) |
| West | 2009 | 5.42 (5.21–5.63) |
| West | 2010 | 5.73 (5.52–5.95) |
| West | 2011 | 6.36 (6.14–6.59) |
| West | 2012 | 6.66 (6.44–6.89) |
| West | 2013 | 6.78 (6.55–7.00) |
| West | 2014 | 7.03 (6.80–7.26) |
| West | 2015 | 7.54 (7.30–7.77) |
| West | 2016 | 7.96 (7.72–8.20) |
| West | 2017 | 8.31 (8.07–8.55) |
| West | 2018 | 8.68 (8.43–8.93) |
| West | 2019 | 8.80 (8.55–9.05) |
| West | 2020 | 13.24 (12.94–13.54) |
| West | 2021 | 16.25 (15.91–16.58) |
| West | 2022 | 13.43 (13.13–13.73) |
| West | 2023 | 12.07 (11.78–12.35) |
| West | **Overall** | 6.91 (6.69-7.14) |

**Supplemental Table 9.** Overall Obesity and Hypertension–related Age-Adjusted Mortality Rates per 100,000 in Adults in the Metropolitan and Non-metropolitan areas in the United States, 1999 to 2020.

| **Age-Adjusted Rate (95% CI)** | | |
| --- | --- | --- |
| **Year** | **Metropolitan** | **Nonmetropolitan** |
| 1999 | 1.34 (1.28–1.39) | 1.21 (1.08–1.33) |
| 2000 | 2.58 (2.49–2.66) | 2.66 (2.48–2.84) |
| 2001 | 2.61 (2.53–2.70) | 2.69 (2.51–2.87) |
| 2002 | 3.00 (2.92–3.09) | 3.34 (3.13–3.54) |
| 2003 | 3.43 (3.34–3.52) | 3.56 (3.35–3.76) |
| 2004 | 3.61 (3.52–3.71) | 3.96 (3.74–4.18) |
| 2005 | 3.85 (3.75–3.94) | 4.47 (4.24–4.70) |
| 2006 | 4.07 (3.97–4.16) | 4.58 (4.35–4.81) |
| 2007 | 4.37 (4.27–4.47) | 4.64 (4.41–4.87) |
| 2008 | 4.64 (4.54–4.74) | 5.31 (5.06–5.55) |
| 2009 | 5.10 (5.00–5.21) | 5.71 (5.45–5.96) |
| 2010 | 5.37 (5.26–5.48) | 5.95 (5.69–6.21) |
| 2011 | 5.89 (5.78–6.01) | 6.68 (6.41–6.96) |
| 2012 | 6.37 (6.26–6.49) | 7.38 (7.09–7.67) |
| 2013 | 6.72 (6.60–6.84) | 7.68 (7.38–7.97) |
| 2014 | 7.19 (7.07–7.31) | 8.43 (8.12–8.74) |
| 2015 | 7.66 (7.53–7.78) | 9.02 (8.70–9.34) |
| 2016 | 8.06 (7.94–8.19) | 9.49 (9.16–9.82) |
| 2017 | 8.31 (8.19–8.44) | 10.06 (9.72–10.39) |
| 2018 | 8.79 (8.66–8.92) | 10.72 (10.37–11.06) |
| 2019 | 9.21 (9.07–9.34) | 11.91 (11.54–12.28) |
| 2020 | 14.23 (14.07–14.40) | 17.22 (16.78–17.66) |
| **Overall** | 5.74 (5.64-5.85) | 6.67 (6.40-6.93) |
